# Comparison of the power between microsatellite and single-nucleotide polymorphism markers for linkage and linkage disequilibrium mapping of an electrophysiological phenotype

**DOI:** 10.1186/1471-2156-6-S1-S7

**Published:** 2005-12-30

**Authors:** Hsiu-Fen Lin, Suh-Hang Hank Juo, Rong Cheng

**Affiliations:** 1 Columbia Genome Center, Columbia University, New York, NY, USA; 2 Department of Epidemiology, Columbia University, New York, NY, USA; 3 Department of Neurology, Kaohsiung Medical University, Kaohsiung, Taiwan

## Abstract

We performed linkage and linkage disequilibrium (LD) mapping analyses to compare the power between microsatellite and single nucleotide polymorphism (SNP) markers. Chromosome-wide analyses were performed for a quantitative electrophysiological phenotype, ttth1, on chromosome 7. Multipoint analysis of microsatellite markers using the variance component (VC) method showed the highest LOD score of 4.20 at 162 cM, near D7S509 (163.7 cM). Two-point analysis of SNPs using the VC method yielded the highest LOD score of 3.98 in the Illumina SNP data and 3.45 in the Affymetrix SNP data around 152–153 cM. In family-based single SNP and SNP haplotype LD analysis, we identified seven SNPs associated with ttth1. We searched for any potential candidate genes in the location of the seven SNPs. The SNPs rs1476640 and rs768055 are located in the *FLJ40852 *gene (a hypothetical protein), and SNP rs1859646 is located in the *TAS2R5 *gene (a taste receptor). The other four SNPs are not located in any known or annotated genes. We found the high density SNP scan to be superior to microsatellites because it is effective in downstream fine mapping due to a better defined linkage region. Our study proves the utility of high density SNP in genome-wide mapping studies.

## Background

Current strategy for complex disease gene mapping usually includes three stages. A genome-wide scan using microsatellite markers is performed to identify interesting chromosomal regions harboring the susceptibility loci. Then fine mapping is used as a follow-up to confirm and narrow the interesting regions. Finally, single nucleotide polymorphism (SNPs) are used to further saturate the regions and discover the candidate genes.

Genetic Analysis Workshop 14 (GAW14) provided data from the Collaborative Study on the Genetics of Alcoholism (COGA), including genome-wide microsatellite markers, genome-wide SNPs and several alcoholism-related phenotypes. This data allowed us to compare the power to detect susceptibility loci between SNPs and microsatellite markers in the context of genome-wide linkage and linkage disequilibrium (LD) analyses. We particularly chose a quantitative electrophysiological phenotype, ttth1 (the data from the Visual Oddball Experiment, measured from far frontal left side channel), as our phenotype of interest because a strong linkage signal was previously detected on chromosome 7 [1]. In this study, we restricted our focus to chromosome 7 rather than a genome-wide search. First, we performed chromosome-wide linkage analysis using microsatellite markers and high density SNPs. We then conducted family-based LD mapping analyses using each single SNP and SNP haplotypes.

## Methods

The COGA data set provided to GAW14 includes 1,350 members with genotype and phenotype information in 143 families. We used the quantitative data of ttth1, microsatellite markers, and two SNP panels (Illumina and Affymetrix panels) on chromosome 7 for our linkage and LD mapping analyses. First, a total of 31 microsatellite markers, at average inter-marker distance of 6.23 cM on chromosome 7, were used for chromosome-wide scan to identify the interesting regions for ttth1. Two-point and multipoint analyses of microsatellite markers were conducted using the variance component (VC) method implemented in the SOLAR [2] and MERLIN programs [3]. Second, the 271 SNPs from the Illumina panel and 578 SNPs from the Affymetrix panel were used for two-point VC analysis using MERLIN. Third, the FBAT program [4] was employed to perform the family-based LD analyses using single SNPs and SNP haplotypes. We used MERLIN to check for recombination between the tightly linked SNPs, and HAPLOVIEW [5] to estimate the linkage disequilibrium statistics (D') as well as the haplotype blocks. SNPs without recombination within haplotype blocks were used to create haplotypes for LD analysis.

## Results

**Figure 1:**
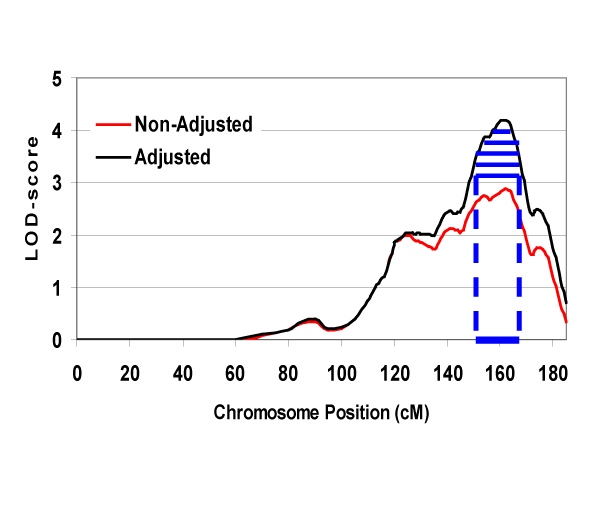
**Microsatellite multipoint VC analysis for ttth1 (chromosome 7). **Microsatellite marker multipoint VC analyses for quantitative trait ttth1 on chromosome 7.

For microsatellite markers, two-point analysis showed the highest LOD score at D7S509 (163.7 cM): unadjusted LOD = 2.70 and age- and sex-adjusted LOD = 3.83. The highest age- and sex-adjusted multipoint LOD score was 4.20 at 162 cM, with a 1-LOD support interval between 150 and 168 cM (Figure 1). The marker closest to the multipoint highest LOD score was D7S509 at 163.7 cM.

**Figure 2:**
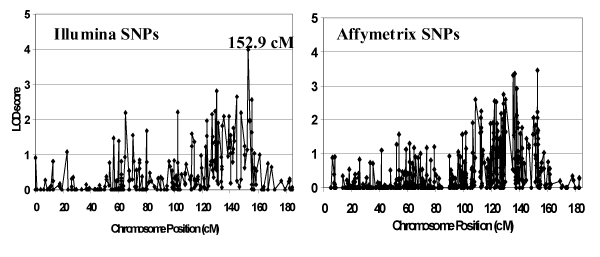
**Age- and sex-adjusted SNP two-point VC analysis for ttth1 on chromosome 7. **Age- and sex-adjusted SNP two-point VC analyses for quantitative trait ttth1 on chromosome 7.

For SNP markers, we found the highest two-point unadjusted LOD score of 3.87, and age- and sex-adjusted two-point LOD score 3.98 at rs940864 (152.90 cM) in the Illumina SNP panel (Figure 2). In the Affymetrix panel, the highest two-point unadjusted LOD score was 2.93, and age- and sex-adjusted two-point LOD score was 3.45 at tsc0063156 (152.94 cM) (Figure 2). Multipoint age- and sex-adjusted analyses were also carried out for Illumina and Affymetrix panels, and the highest LOD scores, 3.70 (152 cM) and 3.13 (151 cM), were found for Illumina and Affymetrix panels, respectively. In either Illumina or Affymetrix panels, the highest LOD score was located within the 1-LOD support interval identified by microsatellite multipoint analysis. Both of the results from the two SNP data sets confirmed that the susceptibility locus is located within the interval of 150–168 cM and the most likely location is approximately 153 cM. The average information contents in the 1-LOD support region are as high as 0.889 and 0.935 for Illumina and Affymetrix SNPs, respectively. Within a 5-cM interval between 150.5 and 155.5 cM, i.e., both sides of the peak SNP LOD scores at 153 cM, there are 23 Affymetrix SNPs and 12 Illumina SNPs. We found 6 Affymetrix SNPs and 5 Illumina SNPs had LOD scores between 1.0 and 1.9, and 4 Affymetrix SNPs and 4 Illumina SNPs had LOD scores >1.9 (data not shown).

Our family-based LD analysis focused on the 1-LOD support interval. A total of 40 Affymetrix SNPs and 24 Illumina SNPs were in this interval. Single SNP LD analyses showed significant associations on three Illumina SNPs and two Affymetrix SNPs (Table 1). Haplotype blocks were generated by using the HAPLOVIEW's default algorithm (confidence intervals). Several haplotype blocks were found in these SNPs with D' ranging from 0.74 to 1.00. The maximum number of SNPs in haplotype blocks was three. In Table 2, we listed only haplotype blocks with significant results. The analyses of haplotypes including 2 or 3 Illumina SNPs, and haplotypes including 2 Affymetrix SNPs showed significant associations. The significant *p*-values for the common (frequency > 0.1) haplotypes ranged from 0.002 to 0.007. The significant haplotype blocks were located at 150.2 cM (rs1476640, rs768055 and rs1859646), 153.036 cM (tsc0058416 and tsc0058418), and 153.912 cM (tsc0590614 and tsc0590615). Comparing the results of the single SNP analysis with SNP haplotype analyses, suggested that the risk-bearing haplotype can be primarily ascribed to one SNP. However, the flanking SNP in the haplotype also contributed additional information leading to a more significant *p*-value. We further searched the SNP database using CHIP Bioinformatics Tools  to find related information of the three closely linked haplotype blocks. The search showed: rs1476640 and rs768055 are located in the introns of the *FLJ40852 *gene, which is a hypothetical protein with unknown gene function, and rs1859646 is located in the intron of the *TAS2R5 *gene, which is a taste receptor. The biological relevance of the three SNPs (rs1476640, rs768055, and rs1859646) were unknown. The other four SNPs (tsc0058416, tsc0058418, tsc0590614, and tsc0590615) are not located in any known or annotated genes.

## Discussion

One of the major advantages of using high-density SNPs over microsatellite markers for genome scans its effectiveness in downstream fine mapping due to a better defined critical region. Our analysis of microsatellite markers showed strong linkage evidence of ttth1 at D7S509 on chromosome 7. However, we could not find significant results for the SNPs near D7S509 (163.7 cM) by either linkage- or family-based LD analysis. Our joint SNP linkage and LD mapping pinpointed a critical region between 150 and 154 cM, which is much smaller than the 1-LOD region by microsatellite markers. Using two different SNP panels, we found that the highest LOD scores and their locations are very close. Using family-based single SNP and SNP haplotype LD analyses, we further identified seven SNPs associated with phenotype ttth1. Our results indicated that the haplotype analysis may be more power than single SNP LD mapping in this dataset. Among them, three SNPs (rs1476640, rs768055, and rs1859646) are located within two potential genes, *FLJ40852 *and *TAS2R5*. It is also noteworthy that the associated SNPs and SNP haplotypes directly under the peak of linkage that is more precisely indicated by SNP markers. Combining linkage and LD analysis approaches, our results suggest that microsatellite markers may be less powerful than SNP markers to indicate the critical region. In our SNP LD analysis, three regions showed association, and there is apparently LD within each region. The strongest LD occurred in the block with two SNPs, tsc0590615 and tsc0590614. A comparison of the two-SNP haplotype LD analysis and the three-SNP haplotype LD analysis did not reveal stronger association in the block of rs1476640, rs768055, and rs1859646. Here, it appears that including more SNPs may not increase the overall evidence for association.

Although MERLIN has the advantage of faster speed than SOLAR in analyzing SNP data, it cannot effectively handle large pedigrees when analyzing microsatellite markers. In this study, we had to increase the default 24 bits to 40 bits while using MERLIN for SNP analysis. In this way, we analyzed all families with MERLIN, but the bit increase is not unrestricted and it may be a problem for even larger pedigree sizes. While we obtained identical results from SOLAR and MERLIN, MERLIN provided results in several hours, while SOLAR required several days.

Three recent studies comparing SNP and microsatellite analysis reported findings similar to ours: high-density SNPs defined a better critical region than microsatellite markers [6-8]. John et al. [6] used both the 10 K Affymetrix SNP panel and 10-cM microsatellite markers to perform a whole-genome screen of multiplex families with rheumatoid arthritis (RA). Their study showed a good concordance between the SNP and microsatellite genome scans. More importantly, the HLA locus, the major RA susceptibility locus on chromosome 6, was better defined by the SNPs than microsatellite markers. Middleton et al. [7] also compared the Affymetrix SNP panel with microsatellite markers in bipolar families. They concluded that a high degree of correspondence existed between the two approaches in general, but that the high-density SNP panel provided more power to detect linkage, especially in regions where the information content and coverage of the microsatellite markers were relatively low and potentially insufficient to detect linkage signal. Similarly, Schaid et al.'s study [8] found that SNP analysis identified more linkage peaks with narrower widths than did microsatellite markers. Moreover, Schaid et al. [8] and Huang et al. [9] also found that multipoint analysis using tightly linked SNPs inflates LOD scores. Therefore, future linkage studies should use SNP without strong LD when performing multipoint analysis.

## Conclusion

This study found that SNP panels provide sufficient meiotic information for linkage analysis. The high-density SNP genome scan is more effective for fine mapping and LD mapping due to a better definition of the linkage region. Multipoint analysis of microsatellite markers showed strong linkage evidence within a 1-LOD support interval from 150 to 168 cM on chromosome 7. Two-point analyses of SNPs showed the highest LOD scores of 3.98 and 3.45 around 153 cM for Illumina and Affymetrix SNP data, respectively. We identified seven SNPs associated with ttth1 in the candidate region harboring potential susceptibility loci using family-based single SNP and SNP haplotype LD analysis.

## Abbreviations

COGA: Collaborative Study of the Genetics of Alcoholism

GAW14: Genetic Analysis Workshop 14

LD: Linkage disequilibrium

RA: Rheumatoid arthritis

SNP: Single-nucleotide polymorphism

VC: Variance component

## Authors' contributions

H-FL performed linkage analyses and summarized the results. RC was responsible for data management, family-based LD analyses and manuscript preparation. S-HHJ designed and oversaw the project and prepared the manuscript. All authors read and approved the final manuscript.

## Figures and Tables

**Table 1:** Family-based single SNP LD analysis

SNP name	Frequency	Map position (cM)	Physical position (bp)	*z*-score	*p*-value	Source
rs1476640	0.448/0.552	150.2	140819587	2.256	**0.024088**	Illumina
rs768055	0.525/0.475	150.2	140820328	2.585	**0.007296**	Illumina
rs1859646	0.413/0.587	150.2	140902881	2.585	**0.009735**	Illumina
						
tsc0058416	0.743/0.257	153.036	144232436	2.073	**0.038212**	Affymetrix
tsc0590615	0.878/0.122	153.912	145141684	2.893	**0.003819**	Affymetrix

**Table 2:** Family-based SNP haplotype LD analysis

Haplotype	Frequency	*z*-score	*p*-value^a^	2 SNPs
h1: 2 1	0.423	2.692	**0.007094**	Illumina's rs
h2: 1 2	0.416	-1.869	0.061690	1476640
h3: 2 2	0.156	-1.175	0.240070	rs768055
h4: 1 1	0.005	-0.761	0.446698	Distance = 741 bp
Global-test	DF = 3	χ^2 ^= 7.564	0.055929	D' = 0.98

Haplotype	Frequency	*z*-score	*p*-value	2 SNPs

h1: 2 1	0.438	-2.325	**0.020064**	Illumina's
h2: 1 2	0.371	3.060	**0.002212**	rs768055
h3: 2 2	0.135	-0.609	0.542501	rs1859646
h4: 1 1	0.056	-0.715	0.474386	Distance = 82,553 bp
Global-test	DF = 3	χ^2 ^= 9.494	**0.023396**	D' = 0.88

Haplotype	Frequency	*z*-score	*p*-value	3 SNPs

h1: 1 2 1	0.379	-2.060	**0.039409**	Illumina's rs1476640
h2: 2 1 2	0.367	3.064	**0.002186**	rs768055
h3: 2 2 2	0.095	-0.981	0.326434	rs1859646
h4: 2 2 1	0.060	-0.633	0.526774	Distance = 741 & 82,553 bp
h5: 2 1 1	0.054	-0.524	0.600176	
h6: 1 2 2	0.039	0.217	0.828502	
Others	0.005	/		
Global-test	DF = 6	χ^2 ^= 10.352	0.110580	

Haplotype	Frequency	*z*-score	*p*-value	2 SNPs

h1: 1 2	0.687	1.402	0.160879	Affymetrix's
h2: 2 1	0.235	-0.318	0.750300	tsc0058416
h3: 2 2	0.048	0.447	0.654974	tsc0058418
h4: 1 1	0.030	-3.456	**0.000549**	Distance = 148 bp
Global-test	DF = 3	χ^2 ^= 12.272	**0.006509**	D' = 0.98

Haplotype	Frequency	*z*-score	*p*-value	2 SNPs

h1: 1 2	0.851	3.097	**0.001958**	Affymetrix's
h2: 2 1	0.115	-1.537	0.124173	tsc0590615
h3: 1 1	0.033	-3.616	**0.000300**	tsc0590614
h4: 2 2	0.001	/		Distance = 14 bp
Global-test	DF = 2	χ^2 ^= 15.661	**0.000397**	D' = 1.00

^a^Bold text indicates significant *p*-value.
